# A Novel Technology for Needle-Free Injections of Liquids, Particles and Viable Cells into the Submucosa of the Urethra by a Pressure-Controlled Waterjet Technology

**DOI:** 10.3390/biomedicines13122986

**Published:** 2025-12-05

**Authors:** Niklas Harland, Andreas Fech, Walter Linzenbold, Bastian Amend, Arnulf Stenzl, M. D. Venkatachalam Rajendran, Markus D. Enderle, Wilhelm K. Aicher

**Affiliations:** 1 Centre for Medical Research, University Hospital, Eberhard-Karls-University, 72072 Tuebingen, Germany; niklasharland86@gmail.com (N.H.); bastian.amend@kliniken-rt.de (B.A.); uro@stenzl.net (A.S.); 2 ERBE Elektromedizin GmbH, 72072 Tuebingen, Germany; andreas.fech@erbe-med.com (A.F.); walter.linzenbold@erbe-med.com (W.L.); markus.enderle@erbe-med.com (M.D.E.); 3 AMET University, Chennai 603 112, Tamil Nadu, India; veerajendran@gmail.com; 4 Department of Thoracic and Cardiovascular Surgery, University Hospital, Eberhard-Karls-University, 72076 Tuebingen, Germany

**Keywords:** needle-free injection of regenerative factors, muscle regeneration, urinary sphincter regeneration, incontinence therapy

## Abstract

**Objectives:** The injection of bioactive compounds into the delicate urethral sphincter muscle to facilitate sphincter regeneration in incontinent patients poses a surgical challenge. In previous preclinical animal studies, approximately half of the 96 pigs treated by transurethral needle injection exhibited misplaced cells or cell loss. We, therefore, investigated whether pressure-controlled waterjet injections delivered nano- and microparticles or liquids more precisely in the urethra and without a risk of full penetration. **Methods:** Fresh cadaveric urethrae were prepared from 12 female pigs. Nano- and microparticles or liquids were injected by waterjet in a proximal (i.e., H5) and distal (i.e., H10) position of the urethral tissue samples employing waterjet pressures of effect 40 (E40), E60, and E80. The injection depths and widths were investigated by histochemistry. **Results:** E40 injections were not sufficient to inject particles into the tissue, while E60 and E80 injections delivered the components into the urethral mucosa, submucosa and close to the urethral muscle. However, employing E80 increased the risk of full penetration of the urethrae. Significant differences in injection depth were not observed between nano- and microparticles. Liquids, however, penetrated the tissue somewhat deeper. Using the optimised pressure protocols facilitated the injection of cells by a novel waterjet prototype with excellent viability into capture fluid. **Conclusions:** Target-specific and pressure-controlled waterjet injections deliver liquids and particles with high precision in the urethra. For future injections of bioactive components, including cells, waterjet injections into the urethrae of incontinent pigs with a pressure of E60 are most promising to investigate the efficacy of regenerative therapies in animal models of urinary incontinence and other diseases or malfunctions.

## 1. Introduction

Urinary incontinence (UI) is a significant and widespread medical challenge, often dismissed as a symptom of ageing that does not require treatment. Throughout an individual’s lifetime, urinary incontinence affects approximately 15% of women and 10% of men [1,2,3]. Others reported higher prevalences for UI [4,5]. With increasing life expectancy, the proportion of patients suffering from this condition also increases [6].

Urinary incontinence has many causes. In women, these are often the long-term effects of birth trauma and pregnancy [7,8]. Obesity, however, also contributes to increased strain and pelvic floor prolapse [9]. Particularly in women, it has been observed that a weakened and asymmetrical contractility of the pelvic floor plays a role in the development of stress urinary incontinence (SUI) [10]. SUI refers to the involuntary loss of urine during physical exertion or increased intra-abdominal pressure without detrusor contraction, such as when coughing or laughing [3]. It is the most common cause of involuntary urine loss in women. But changes in tissue composition, such as the loss of muscle and nerve cells, also play a role in its development.

In men, the primary cause of urinary incontinence is the sequela of surgical resections for benign prostatic hyperplasia and prostate carcinoma [11,12]. Despite a recent decline in the incidence of prostate cancer in industrialised countries [13], prostate cancer is still among the most common malignancies, and the second most common cancer worldwide [14]. While radical resection operations or even complete prostatectomies are still the standard of treatment, severe damage to the surrounding tissue, including nerve and vascular pathways, may occur, despite ever-improving surgical techniques [15]. The fibrosis of the prostate itself, as well as iatrogenic tissue damage and fibrosis of the urethral sphincter muscle, may contribute to incontinence [16]. Since the problem is often exacerbated by subsequent transurethral surgical interventions, considerable hope is placed in tissue engineering and stem cells, specifically in the functional regeneration of the sphincter using biological tools and the body’s own healing mechanisms [3,17,18,19].

Nerve damage caused by radiation or other nerve damage also plays a role in SUI aetiology [20,21,22]. However, the structural changes in the irradiated tissue are a process that occurs over a long period of time. Atrophic muscle cells are replaced by connective tissue [23]. The urethral sphincter apparatus thus loses its contractile function. Moreover, the external sphincter muscle has no bony anchorage points. It derives its stability solely from its close connection with the pelvic floor muscles and the *musculus levator ani.* The sphincter dysfunction can, thus, occur due to the relaxation and weakening of the surrounding tissue as well.

Current SUI therapies include management of liquid uptake, pharmacological strategies, physiotherapy, and electrostimulation of the sphincter muscle [24,25,26]. If these treatments fail to yield success, surgical interventions may be considered. These include injections of bulking agents to strengthen the atrophic tissue in the lower pelvic floor and insertion of vaginal tapes or slings [27,28]. However, such therapies inherit their specific adverse effects and may not provide long-lasting benefits, as different studies reported rather moderate success [29,30,31]. On the other hand, injection of tissue regenerative cells may lead to a persistent improvement of the SUI. This has been investigated in many preclinical and clinical studies, albeit with variable and sometimes unconvincing results [3,17,18,32,33,34,35,36,37,38].

In our preclinical SUI model, we detected the injected cells only in 45% of the animals in the urethra, in the submucosal, mucosal or muscular tissue layers [39]. Often, full penetrations of the urethrae by the injection needle were observed. In some samples, no cells were detected [39]. Thus, we concluded that transurethral needle injections of cells or other medications in the sphincter complex carry a significant risk of malplacement of the therapeutic compounds. This malplacement of the bioactive components could be one reason for the variable results of previous SUI cell therapy studies [3,17,18,32,33,34,35,36,37,38]. In addition, injections of bioactive components in delicate tissues by needle may cause local injury, as needles punch holes in solid tissue. Moreover, moving the needles injected in a tissue sidewise may cause detrimental cuts. Such damage may contribute to a loss of the active components to be applied. In contrast, injections by waterjet do not require the penetration of the tissue targeted. Here, the injection device is only placed onto the tissue, but it does not penetrate it. Only the narrow waterjet penetrates the tissue. Thus, we hypothesised that replacing needle injections by the novel waterjet technology might reduce the risk of tissue injury, including full penetration of the urethra and, at the same time, might improve the target accuracy of the injection. The objectives of this study were to investigate, (i) the pressure range required to transport liquids and particles through the urothelial layer and the submucosal tissue close the sphincter muscle; (ii) if a pressure-controlled waterjet technology facilitated a precise injection of liquids, fluorescent nanoparticles (fNPs, ø 560 nm), resembling small drugs such as slow-release complexes, or fluorescent microparticles (fMPs, ø = 20 μm), resembling the sizes of mesenchymal stromal cells, fibroblasts, myoblasts or other regenerative cells into the porcine urethra, and (iii) the pressure maxima tolerable in the urethra to avoid a full penetration of the urethra by the jet.

Beforehand, cells were injected through tubing of different calibres under varying pressure levels to identify the optimal tubing calibres and corresponding parameters that preserve cell viability. The porcine model was chosen for two main reasons: (i) it allows 1:1 scale surgical procedures in future in vivo studies using the same prototypes, instruments, and technologies as in clinical practice; and (ii) unlike commonly used models such as dogs or rodents [33,40,41]—pigs share close histological similarities with humans in the urethral closure complex [42,43,44,45]. Moreover, the pig urethral sphincter muscle predominantly consists of slow-twitch muscle fibres [46].

In this exploratory study, we delineate the pressure levels required to inject fluids, nano- and microparticles through the urothelium into the urethral tissue without a significant risk of full penetration. Further optimisation of this pressure profile allowed the injection of highly viable cells by waterjet. This new method can now be employed in future studies in a living SUI model to determine its efficacy for future incontinence therapies.

## 2. Materials and Methods

### 2.1. Investigation of the Calibre of Tubings as a Function of the Pressure Levels Required to Grant Sufficient Cell Viability After Waterjet Injections

To determine the viability of cells transported through injection device depending on the pressure applied and as a function of the calibre of the tube, MonoMac 6 cells and NIH3T3 cells were expanded as requested by the supplier (ATCC; Manassas, VA, USA), harvested by mild proteolysis (Trypsin-EDTA, Sigma-Aldrich; Taufkirchen, Germany), washed by DMED and counted by Trypan Blue dye exclusion (Sigma-Aldrich) and a hematocytometer. Only batches with a starting cell viability above 95% were included in the experiments. To determine the diameter of MonoMac6 and NIH3T3 in suspension, the cells were harvested and their sizes were measured in suspension by an automated cell counter and analyser device (CASY, Schärfe Systems; Reutlingen, Germany) and confirmed by laser scanning microscopy (LSM 510; Zeiss, Oberkochen, Germany) and proprietary image analysis software (ZEN 2.5, Zeiss, Oberkochen, Germany). For waterjet injections, the cells were resuspended in DMEM medium and complemented with either gelatin (Sigma-Aldrich), Albugel^®^ (TETEC; Reutlingen, Germany), or fetal bovine serum (Sigma-Aldrich) in different concentrations. The cells were injected with pressures ranging from E10 to E80 through metal tubes with and without nozzles. The calibres of the tubes included ranged from 160 μm to 500 μm at a length of 30 cm. After loading the cells in the dosing unit of the injection device, the cells were injected into centrifugation tubes filled with 5 mL cell culture media as capture fluid, sedimented by centrifugation (200× *g*, 10 min, 4 °C), and resuspended in 1 mL medium. The yield and the viability of the cells were determined by brightfield microscopy (Axiovert A1, Zeiss) using a hematocytometer and the Trypan Blue dye exclusion method and confirmed in some cases by an automated cell counting device as described by the supplier (CASY).

### 2.2. Preparation of Urethral Tissue Samples and Waterjet Injection

**Figure A1:**
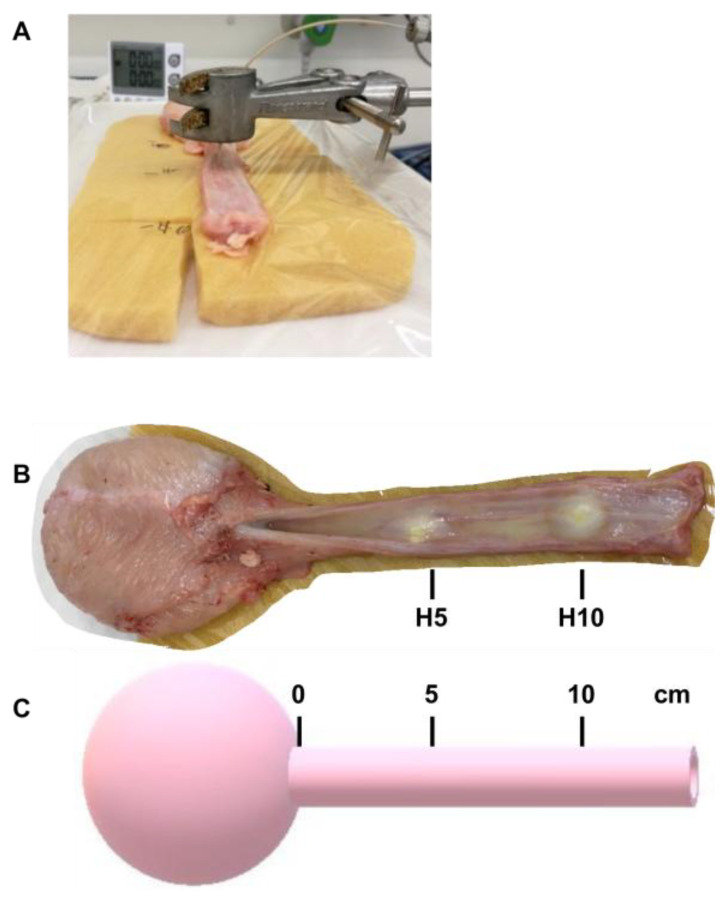
In vitro injection in cadaveric tissue samples and injection positions. (**A**) Fresh cadaveric urethral samples were prepared from female pigs and placed on an elastic sponge to mimic the tissue elasticity of the lower pelvic floor. Using a laboratory clamp, the injection tube was precisely placed on the tissue. Particles (fNPs, fMPs) or liquids were applied by waterjet using different pressure profiles. (**B**) Top view of a porcine cadaveric urethra after injection of fluorescent particles in positions H5 and H10 as indicated. (**C**) Schematic view for determining the positions H5 and H10 of porcine urethral samples.

The urinary bladder and urethra samples were prepared from twelve fresh cadaveric female pigs obtained from a local slaughterhouse. Surrounding adipose and connective tissues were removed. A catheter was inserted to open the urethra by a longitudinal cut using scissors (Figure A1). The opened urethra was placed on plastic wrap on a foam pad, which reproduced the elasticity of the periurethral tissue in situ. To avoid drying out, the tissue was moistened with PBS. The injection device was lowered exactly onto the inner surface of the urothelium by a sample holder and microgauge device, and connected to a waterjet device (ERBEJET2, Erbe Elektromedizin GmbH; Tuebingen, Germany; Figure A1A). Each urethra was injected twice with the same pressure setting employing a proximal and a distal position (Figure A1B). Bioinert fluorescent nanoparticles (fNPs, ø = 560 ± 10 nm) and microparticles (fMPs, ø = 20 ± 0.4 μm; PS fluogreen, micro particles; Berlin, Germany) were resuspended in PBS (1:10 *v*/*v*). The fNPs, fMPs were injected by vertical waterjet 5 cm (H5) and 10 cm (H10) distal from the bladder neck (Figure A1B,C). Injections of an isotonic liquid (Tissue Marking Dye, Triangle Biomedical Sci.; Durham, NC, USA) served as controls. The waterjet pressure settings were E10 to E80, and the injection volumes were set to 500 μL per injection.

### 2.3. Preparation of Cryosections and Histologic Evaluation

The areas of injection (Figure A1) were cut out of the urethra by a scalpel and scissors, submerged in cryomolds, covered with freezing liquid (FS 22 frozen section media; Leica Biosystems, Wetzlar, Germany), slowly submerged and stored in liquid nitrogen. Cryosections (30 μm) were generated (CM 1860 UV, Leica Microsystems; Mannheim, Germany), mounted on glass slides (SuperFrost Plus, Langenbrinck; Emmendingen, Germany), and stained by the AZAN method (Carl Roth; Karlsruhe, Germany) to visualise the submucosal and mucosal connective tissue in blue and muscular tissue in red colours, respectively. The stained cryosamples were observed by microscopy (Axiovert 200M with Axiocam HRc, Zeiss) and recorded (AxioVision V 4.8.2.0; Zeiss). Individual micrographs were stitched together to generate overview images using the Image Composer Editor software (V2.0.3.0; Microsoft, Redmont, WA, USA)). The distance of the particle clusters and dimensions of the injected liquids were measured with the proprietary software of the microscope (AxioVision, Zeiss) and processes using a spreadsheet software (MS Excel, V16.103.2, Microsoft).

### 2.4. Preparation of Porcine Stromal Cells and Myoblasts for Waterjet Injections

**Figure A2:**
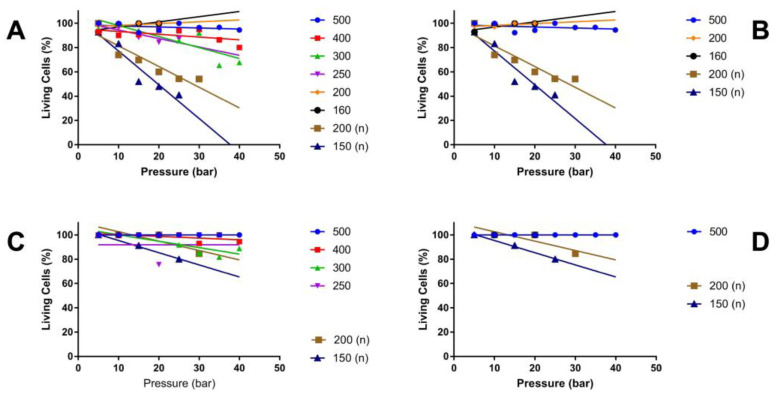
Relationship between cell vitality, injection pressure and pipe width after waterjet injection. MonoMac 6 (**A**,**B**) or NIH3T3 (**C**,**D**) were injected in capture fluid, and the viability of the cells was enumerated as a function of the injection pressure and calibre of the pipes. Calibres, i.e., inner diameters, of 160 μm to 500 μm were included as indicated. Pipes with a nozzle are marked by (n). The viability of the cells is denoted on the *y*-axis in per cent, and the pressure levels applied on the *x*-axis (bar).

Adipose tissue-derived mesenchymal stromal cells (ADSCs) and myogenic progenitor cells (MPCs) were isolated from pig cadaveric subcutaneous fat tissue or *M. extensor carpi radialis,* respectively, expanded, and characterised as described [47,48,49]. Upon reaching a confluence of approximately 70%, cells were harvested as described above, and their viability was enumerated. Based on our preparatory study (Figure A2), the ADSCs were diluted to 5 × 10^6^/mL and the MPCs were diluted to 2.5 × 10^6^/mL in their corresponding expansion media and injected by waterjet in 100 μL aliquots in 5 mL cell culture medium using the E10–E60 pressure settings. The injected cells were sedimented by centrifugation (7 min, 300× *g*, 20 °C), the supernatant was aspirated, the cells were resuspended in 1 mL PBS, and the yield and viability were counted as described above.

### 2.5. Data Processing

Numerical experimental results were transferred to a spreadsheet program (Excel, V16.102.3; Microsoft), sorted, and calculated. The graphics were created using Excel or Prism 7.0 (GraphPad Software; Boston, MA, USA).

## 3. Results

### 3.1. Determining the Calibre of the Waterjet Injection Devices for Cell Injections

The mean diameter of MonoMac6 and NIH3T3 cells in suspension was determined by CASY and LSM at 19 to 21 μm. To avoid unspecific cluster formation of cells in narrow pipes, tubes with calibres of 160 μm to 500 μm were utilised to inject the cells in capture fluid and to determine the cell viability. The pressure settings employed varied between E5 and E40 (Figure A2). Using MonoMac6 cells, viabilities above 80% were computed with open calibres above 400 μm over the full range of pressure applied. At lower pressure levels of E5 or E10, 80% viability was achieved with open tubes as narrow as 160 μm of inner diameter (Figure A2A,B). Adding a nozzle to the injection tube and, thus, changing the spray characteristics of the jet, reduced the viability of cells even at a pressure level of E10 to the minimally acceptable viability level of 80%, and the cell viability remained below this level at higher pressure levels (Figure A2A). A second series of experiments with MonoMac6 confirmed these results (Figure A2B). To avoid bias by the cell line selected, we repeated these experiments with NIH3T3 cells (Figure A2C,D). Almost 100% cell viability was observed with wide tubes of 500 μm and 400 μm inner diameter (Figure A2C). Tubes with a calibre of 300 μm granted cell viabilities of NIH3T3 above 80% up to pressure levels of E40, while injections of cells through tubes as narrow as 250 μm reduced the viability of NIH3T3 cells (Figure A2C). As NIH3T3 cells seemed more robust against shear stress when compared to MonoMac6 cells, we repeated the experiments with these cells and used rather narrow tubes with nozzles (Figure A2D). With NIH3T3 cells, sufficient cell viability was recorded at pressure levels of E20 to E30 (Figure A2D). The results indicated that the sensitivity to mechanical stress, such as shear stress, differs between cell types. To facilitate future injections with different types of cells, depending on the clinical need, and at the same time to be capable of adapting the design of the injection device to the surgical requirements, cell injections were performed in the future injection experiments with a maximal pressure level of E10. Moreover, to ensure that this injection method could be used in the future not only with wide endoscopes, such as those used in GI surgeries, but also in minimally invasive applications in urology, gynaecology, or other disciplines that use endoscopes with smaller working channels, the technology has been further developed with calibres below 250 μm.

### 3.2. Determining the Pressure Levels Required to Transport the Particles in the Urethral Tissue

**Figure A3:**
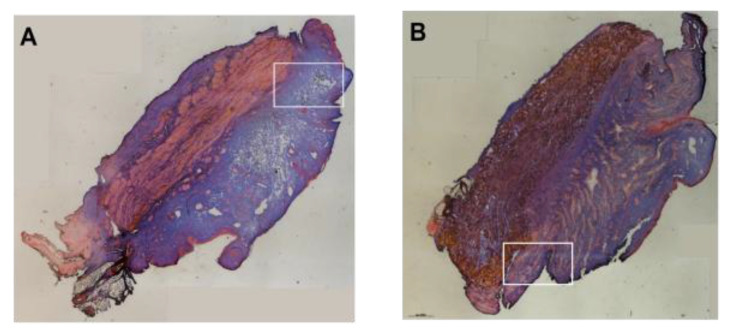
Analysis of particle injections with low-pressure profiles. Fluorescent microparticles were injected in urethral tissue samples at positions H5 (**A**) or H10 (**B**) by a low-pressure of E20. The fMPs barely passed the urethra (white open rectangles). Injection bubbles in the mucosal connective tissue or stretching of the sphincter muscle were not observed. An inflow of liquid was not observed.

To investigate the minimal pressure levels required to transport liquids or particles, including cells, across the ureothelium and in the tissue of the urethra, fMPs were injected by waterjet in fresh cadaveric tissue samples (Figure A3). Using low pressure levels of E5 to E20 failed to inject particles into the tissue at the positions H5 and H10 of the porcine urethra. Applying a pressure level of E40 injected the fMPs just across the urothelial layers, indicating that pressure levels above E40 were required to transport particles deeper into the porcine urethra and close to the sphincter muscle (Figure A3). Thus, for the time being, waterjet injections of fNPs and fMPs were continued with pressure levels of E60 to E80.

### 3.3. Injections of Nanoparticles in the Porcine Urethra by Waterjet with Moderate Pressure E60

**Figure 1:**
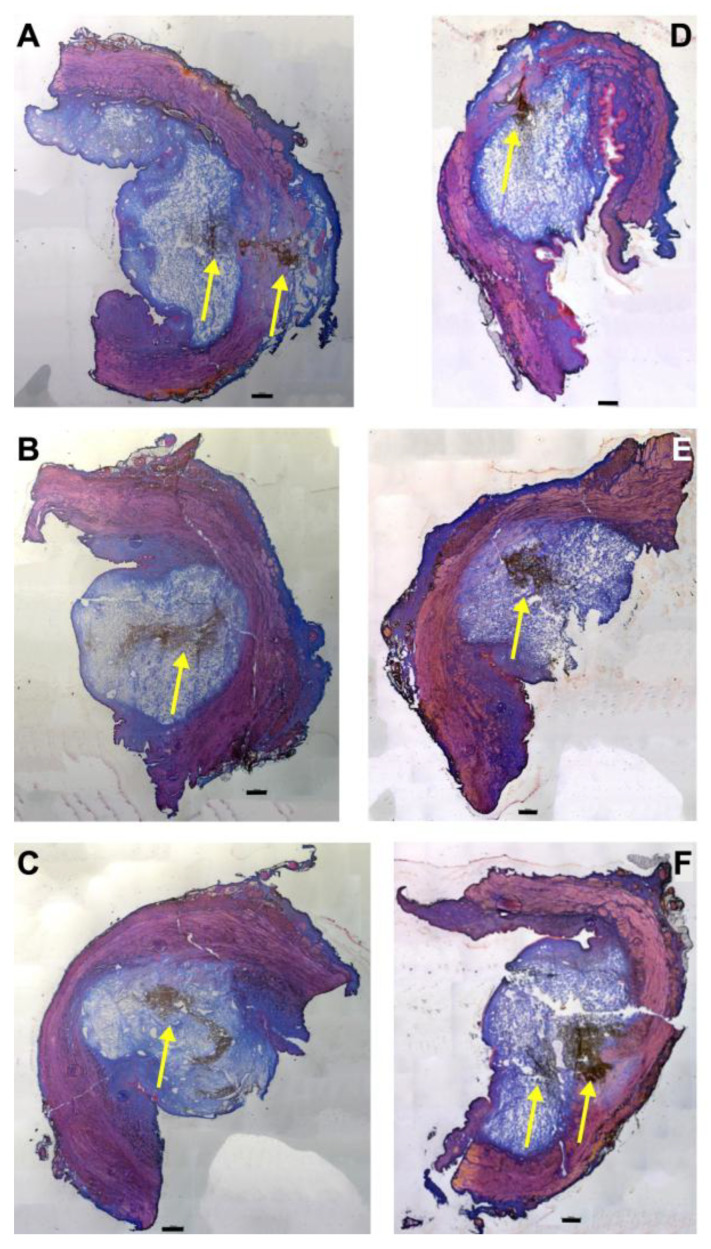
Injection of nanoparticles in the urethral tissue by a pressure setting of E60. The fNPs were injected by waterjet at pressure settings of E60 in urethral tissue samples 5 cm below the bladder neck (H5; (**A**–**C**)) and 10 cm below the bladder neck (H10, (**D**–**F**)). Cryosections were generated and stained with AZAN. The injections distended the connective and muscular tissues, as indicated by the light blue bubble in the connective tissue. The sphincter muscle appears red. The injected fNPs are recorded as clusters of dark spots (yellow arrows). The figure displays representative examples of injections in the urethrae of 6 pigs. Size bars indicate 1000 μm.

Fluorescent nanoparticles (fNPs) were injected into cadaveric porcine tissue samples using a pressure setting of E60. The waterjet injections generated bubble-like artefacts in the submucosal and mucosal layers corresponding to the volume injected (Figure 1). Using E60, full penetrations were not observed when injecting the fNPs approximately 5 cm (H5) or 10 cm (H10) distal of the bladder neck (Figure 1, Figure A1). In some samples, the fNPs were detected in or at the sphincter muscle (Figure 1A,D,F), while in other samples the fNPs were detected in the submucosa and mucosa tissue (Figure 1B,C,E).

**Figure 2:**
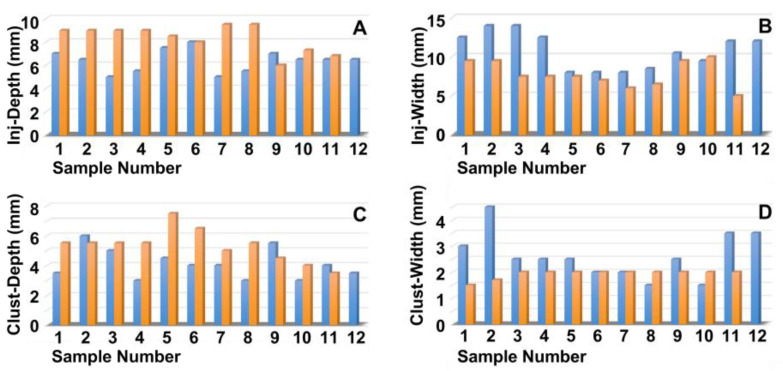
Measuring the positions of the nanoparticles in the tissue targeted. The fNPs were injected by E60 pressure settings at positions H5 (blue bars) and H10 (orange bars), and the injection depth was determined as the distance of the fNPs from the urethral lumen (**A**), the injection width in the tissue (**B**), the depths of the fNP clusters in the tissue in the direction of injection (**C**), as well as the cluster widths in the tissue (**D**) were determined by bright field microscopy of cryosections and measured by the proprietary software of the microscope. The *x*-axis presents the tissue samples, and the *y*-axis the dimensions in mm as indicated.

**Figure 3:**
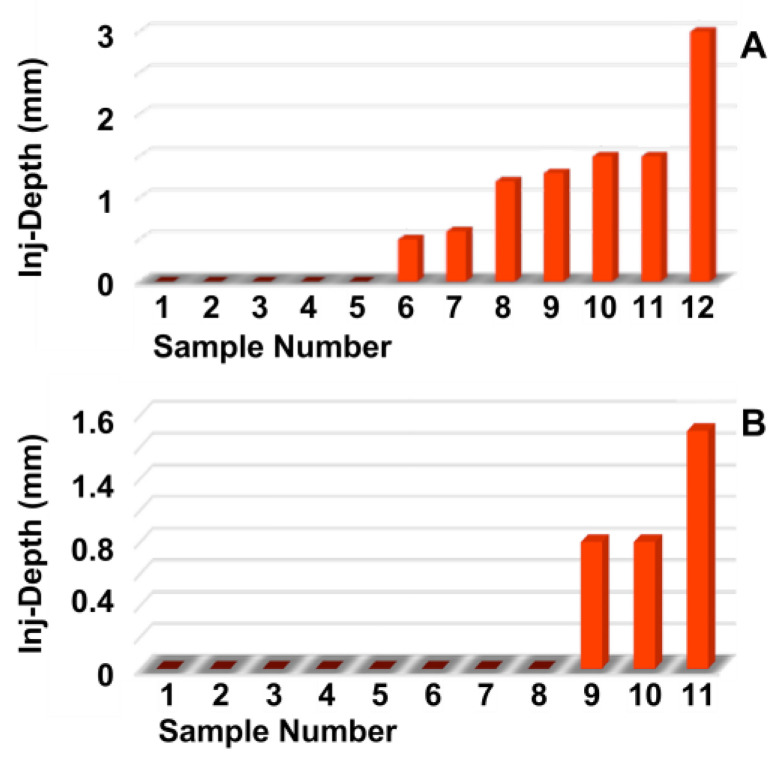
Determining the injection depths of nanoparticles in the sphincter muscle. The fNPs were injected by E60 pressure settings at positions H5 (**A**) and H10 (**B**). The injection depth relative to the sphincter muscle was determined by bright field microscopy of cryosections and measured by the proprietary software of the microscope. The *x*-axis presents the tissue samples, and the *y*-axis the dimensions in mm as indicated.

Next, the penetration depth and width of the particle suspension in the mucosa (Figure 2A,B), as well as the dimensions of the fNP clusters (Figure 2C,D), were enumerated after E60 injections of fNPs into positions H5 and H10, respectively. At position H5 (blue bars), the mean penetration depth of the suspensions was 6.3 mm, and the mean penetration width 10.6 mm (Figure 2A,B). The particle clusters were detected in an area 4.1 mm deep and 2.6 mm wide (Figure 2C,D). At position H10 (orange bars), a slightly deeper but narrower penetration of the fNPs was noted. The mean penetration depth of the suspensions was 8.3 mm, and the penetration width 7.8 mm (Figure 2A,B). The fNP clusters were detected in an area 4.8 mm deep and 1.6 mm wide (Figure 2C,D). In addition, the approach of the particles to the sphincter muscle was investigated (Figure 3). At position H5, in seven out of the twelve urethrae employed, fNPs penetrated the sphincter muscle up to 3 mm (mean 1.35 ± 0.8 mm; Figure 3A). In contrast, at position H10, in only three of the eleven urethrae employed, a muscle penetration was noted and the mean penetration depth was lower (1 ± 0.4 mm; Figure 3B). This difference in the penetration depth of the fNPs in the sphincter muscle observed between H5 and H10 injections is in line with the differences in sphincter muscle composition and muscle strength observed in pigs [46,50].

### 3.4. Injections of Nanoparticles in the Porcine Urethra by Waterjet with Elevated Pressure E80

**Figure 4:**
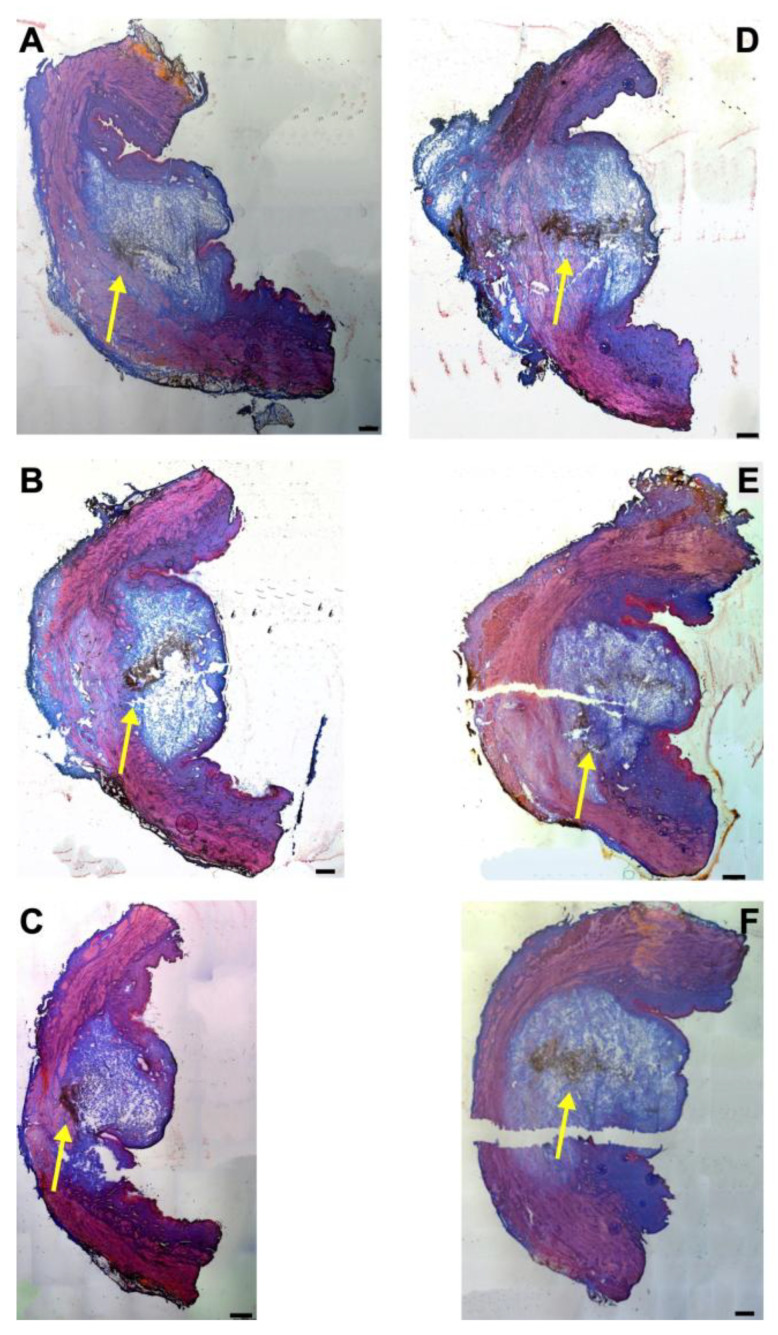
Injection of nanoparticles in the urethral tissue by a pressure setting of E80. The fNPs were injected by waterjet at pressure settings of E 80 in urethral tissue samples 5 cm below the bladder neck (H5; (**A**–**C**)) and 10 cm below the bladder neck (H10, (**D**–**F**)). Cryosections were generated and stained with AZAN. The injections distended the connective and muscular tissues, as indicated by the light blue bubble in the connective tissue. Muscle tissue appears red. The injected fNPs are recorded as clusters of dark spots (yellow arrows). The figure displays representative examples of injections in the urethrae of six pigs. Size bars indicate 1000 μm.

Next, the fNPs were injected in cadaveric porcine tissue samples using a pressure setting of E80 (Figure 4). As observed upon E60 injections (Figure 1), the waterjet injection caused a swelling of the submucosal and mucosal tissue reflecting the injected volume (Figure 4). Upon injections at E80, a slightly more prominent stretching of the sphincter muscle was noted in both, the H5 (Figure 4A–C) and H10 regions (Figure 4D–F).

**Figure 5:**
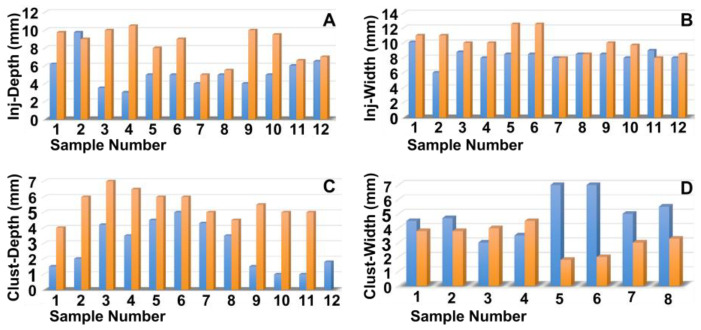
Measuring the positions of the nanoparticles in the tissue targeted. The fNPs were injected by E80 pressure settings at positions H5 (blue bars) and H10 (orange bars) and the injection depth determined as the distance from the urethra lumen (**A**), the injection width in the tissue (**B**), the depths of the fNP clusters in the tissue in the direction of injection (**C**), as well as the cluster widths in the tissue (**D**) were determined by bright field microscopy of cryosections and measured by the proprietary software of the microscope. The *x*-axis presents the tissue samples, and the *y*-axis the dimensions in mm as indicated.

**Figure 6:**
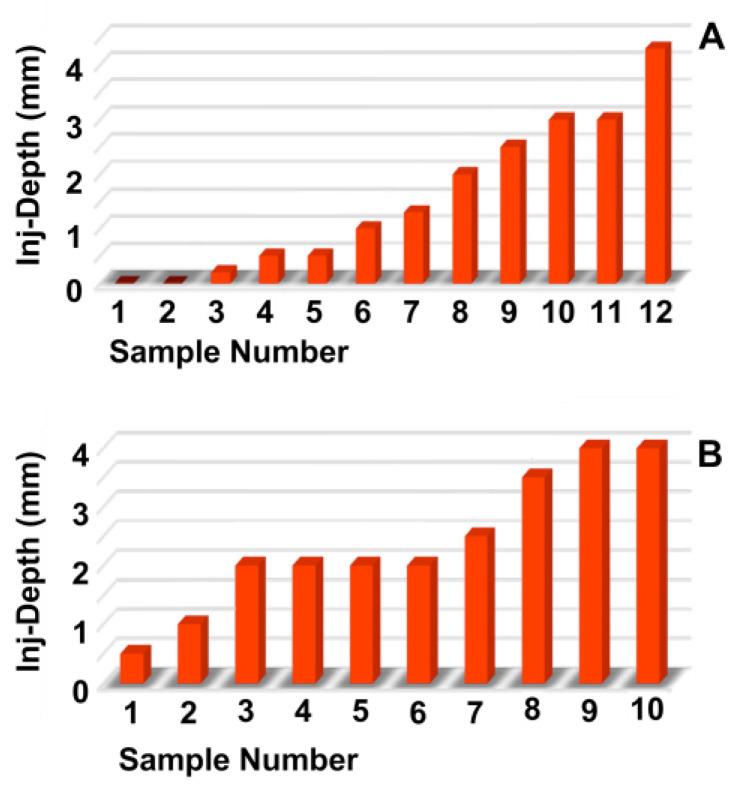
Determining the injection depths of nanoparticles in the sphincter muscle. The fNPs were injected by E80 pressure settings at positions H5 (**A**) and H10 (**B**). The injection depth relative to the sphincter muscle was determined by bright field microscopy of cryosections and measured by the proprietary software of the microscope. The *x*-axis presents the tissue samples, and the *y*-axis the dimensions in mm as indicated.

**Figure A4:**
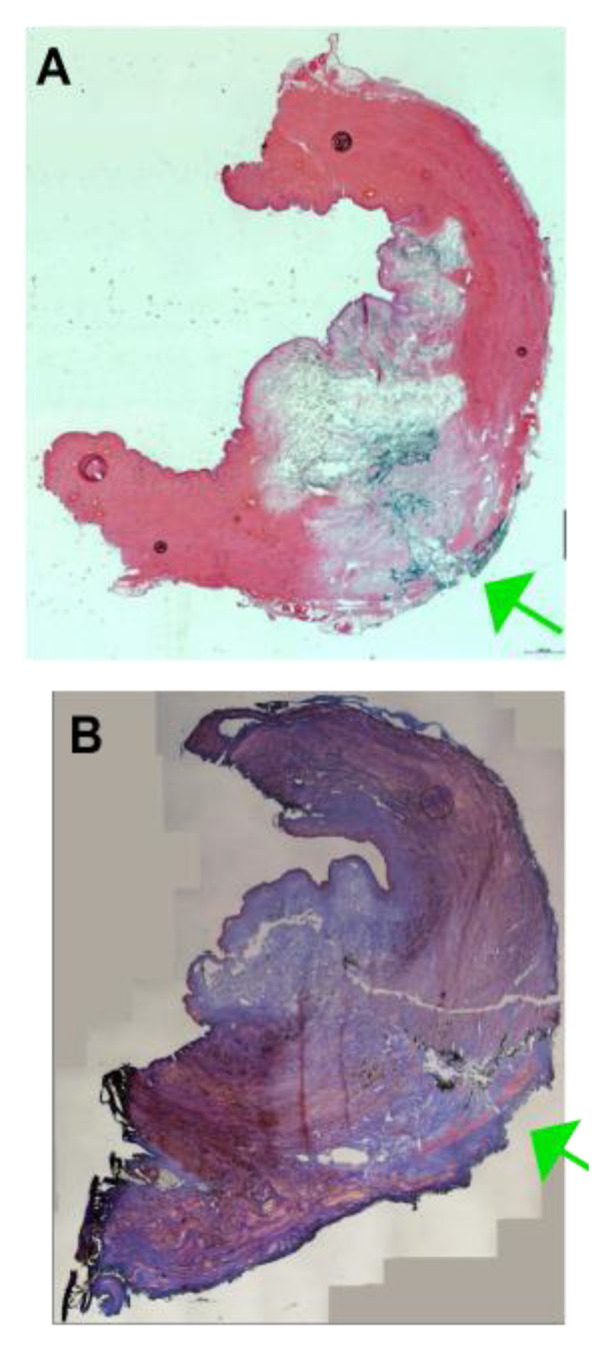
Examples of full penetrations of the urethra upon injection with elevated pressure profiles. The fNPs (**A**) or fMPs (**B**) were injected in the porcine urethra using an elevated pressure profile of E80. In some cases, a full penetration of the jet was observed, and the fluorescent particles were recorded throughout the sphincter muscle and on the outside of the samples (green arrows).

The penetration depth and width of the particle suspension in the mucosa, as well as the dimensions of the fNP clusters, were enumerated after E80 injections of fNPs into position H5 and H10 (Figure 5). At position H5 (blue bars), the mean penetration depth of the suspensions was 5.2 mm, the width 8.3 mm (Figure 5A,B). The particle clusters were detected in an area 2.8 mm deep and 1.7 mm wide (Figure 5C,D). At position H10 (orange bars), a comparable penetration of the fNPs was noted using the E80 profile: The mean penetration depth of the suspensions was 8.2 mm, the width 9.8 mm (Figure 5A,B). The fNP clusters were detected in an area 5.5 mm deep and 2.3 mm wide (Figure 5C,D). In addition, the approach of the fNPs to the sphincter muscle was investigated (Figure 6). At position H5, in ten out of the twelve urethrae employed, fNPs penetrated the sphincter muscle up to 4.3 mm (mean 1.82 ± 1.35 mm; Figure 6A), while the fNPs remained in the connective tissues in two samples. At position H10, an elevated muscle penetration was noted in ten of the twelve samples investigated (2.35 ± 1.17 mm; Figure 6B). A full penetration and, thus, a loss of fNPs was observed in two of the twelve samples employed (Figure A4A).

### 3.5. Injections of Microparticles in the Porcine Urethra by Waterjet with Moderate Pressure E60

**Figure 7:**
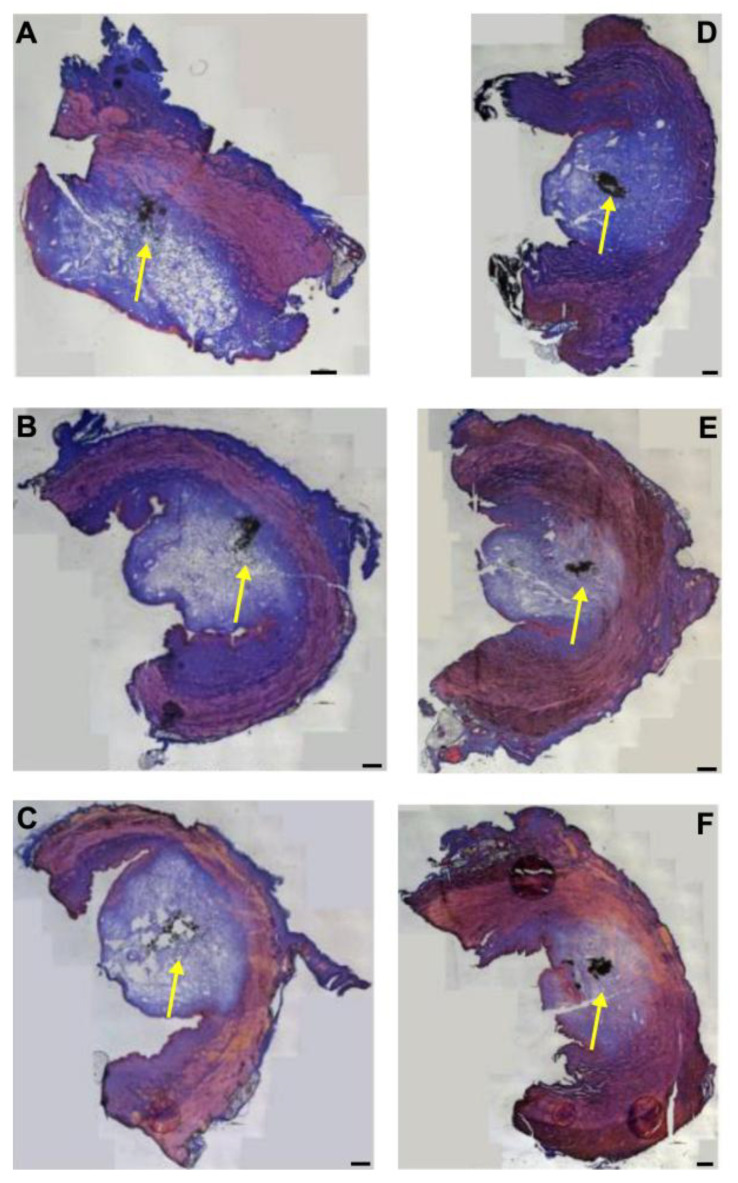
Injection of microparticles in urethral tissue by a pressure setting of E60. The fMPs were injected into the tissue samples 5 cm below the bladder neck (H5; (**A**–**C**)) and 10 cm below the bladder neck (H10, (**D**–**F**)), and cryosections were generated and stained by AZAN. The waterjet injections distended the connective and muscular tissues, as indicated by the light blue bubble in the connective tissue. The injected fNPs are recorded as clusters of dark spots (yellow arrows). The figure displays representative examples of injections in the urethrae of six pigs in total. Size bars indicate 1000 μm.

Fluorescent microparticles (fMPs), resembling the size (ø ≈ 20 μm) and density (r ≈ 1.07 g/mL) of cells, were injected into cadaveric porcine tissue samples as well. At first, a pressure setting of E60 was employed (Figure 7). As seen with fNPs, the liquid injected with an E60 pressure level inflated a tissue bubble mainly in the mucosal and submucosal layers. An evident stretching of the sphincter muscle was not observed (Figure 7). At positions H5 (Figure 7A–C) and H10 (Figure 7D–F), the 20 μm fMPs reached in some samples the sphincter muscle layer (Figure 7A,B,E,F), in others the fMPs were found in the mucosal layer (Figure 7C,D). Applying a pressure level of E60, a full penetration of the urethral samples was not observed (Figure 7).

### 3.6. Injections of Microparticles in the Porcine Urethra by Waterjet with Elevated Pressure

**Figure 8:**
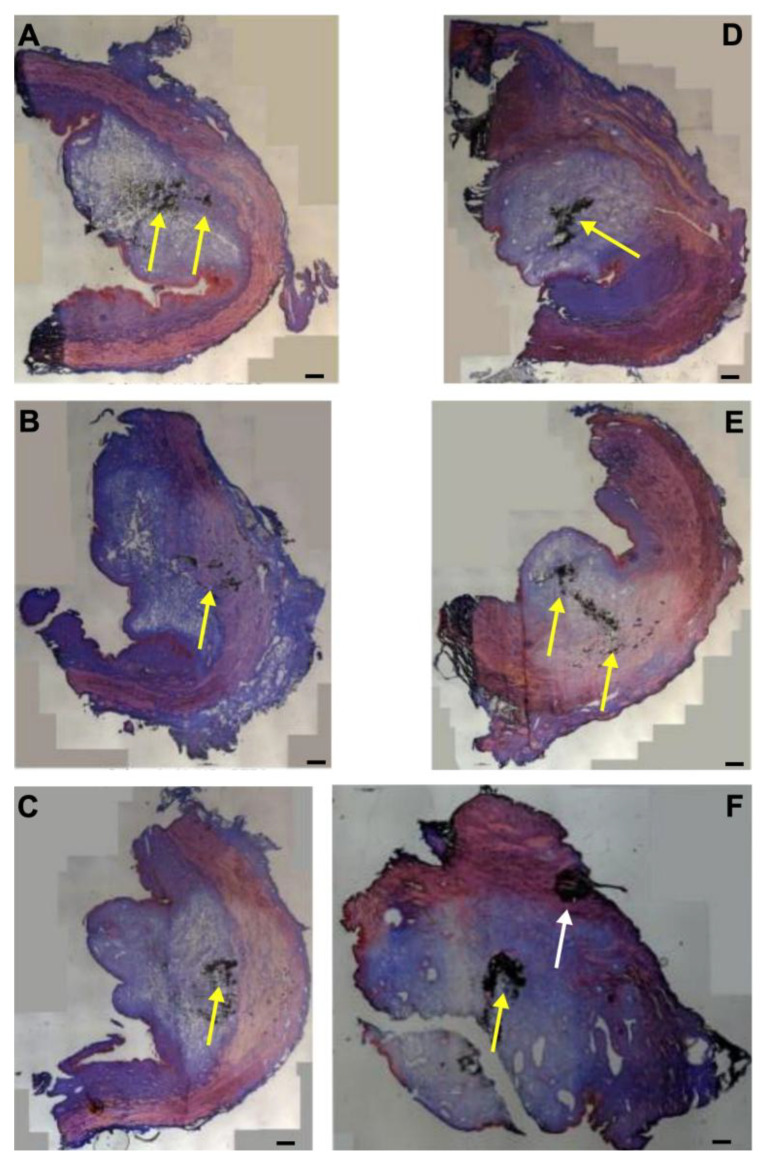
Injection of microparticles in the urethral tissue by a pressure setting of E80. The fMPs were injected into the tissue samples with the elevated pressure profile E80 5 cm below the bladder neck (H5; (**A**–**C**)) and 10 cm below the bladder neck (H10, (**D**–**F**)), and cryosections were generated and stained by AZAN. The injections distended the connective and muscular tissues. The injected fNPs are recorded as clusters of dark spots (yellow arrows). The black cluster marked in F by a white arrow is considered an artefact as in the surrounding tissue no signs of tissue stretching or liquid injections are observed. The figure displays representative examples of cryosections of the injection sites of the urethrae of six pigs in total. Size bars indicate 1000 μm.

Next, fMPs were injected in porcine urethra samples with an elevated pressure of E80 by waterjet (Figure 8). In some samples, the fMPs were found in the mucosal and submucosal tissue of the fresh cadaveric porcine urethra samples, in others close to or even in the sphincter muscle (Figure 8). As observed upon injections with fNPs at pressure levels of E80, some samples presented with stretching of the sphincter muscle (Figure 8C,E), others even with full penetration through the sphincter muscle (Figure A4B). In some samples, the fMPs reached the sphincter muscle (Figure 7A–C,E,F) while in others they remained in the mucosal layer (Figure 7D).

### 3.7. Injections of Isotonic Liquids in the Porcine Urethra by Waterjet

**Figure 9:**
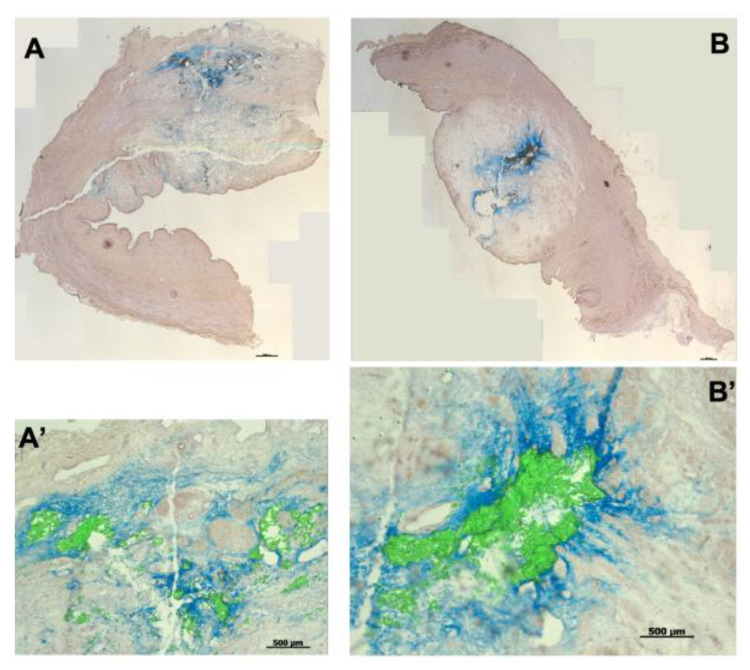
Injection of microparticles and isotonic ink in porcine urethral tissue samples. Fluorescent microparticles and tissue ink were mixed 1:1 and injected at positions H5 (**A**) and H10 (**B**) in the urethral tissue samples using E60 pressure settings. Cryosections were generated and stained with HE. The ink penetrated somewhat deeper in the tissue compared to fMPs. (**A’**,**B’**) present enlarged image sections. The size bars in (**A**,**B**) represent 1000 μm, in (**A’**,**B’**) 500 μm, respectively.

Waterjet injections using pressure levels of E60 seemed most promising for future studies investigating the regenerative capacities of bioactive compounds for urethral sphincter regeneration. At E60, an obvious compression or stretching of the sphincter muscle was not evident at positions H5 and H10, and full penetrations of the jet were not observed. Thus, we compared the injection depth of 20 μm fMPs with the injection depth of the ink methylene blue in cadaveric urethra tissue samples (Figure 9). At both positions, H5 and H10, the fMPs were found in the same areas as the injected ink. The ink, however, penetrated further into the tissue and formed a kind of rim around the injected particles (Figure 9). However, the overall positions of the injected liquid and the fMPs were not different.

### 3.8. Injection of Porcine Stromal Cells and Myoblasts by Waterjet

**Figure 10:**
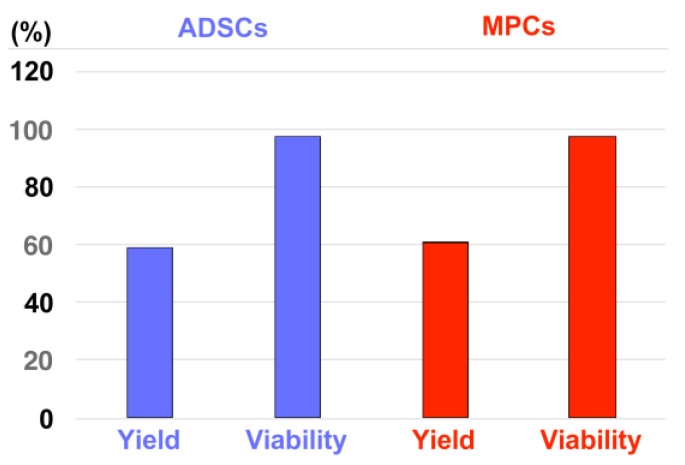
Injection of living porcine cells with the novel injection device in capture fluids. Porcine ADSCs (starting density: 5 × 10^6^/mL, 4 injections, blue bars) and MPCs (starting density: 2.5 × 10^6^/L, 12 injections, red bars) were injected in capture fluid by a novel waterjet injector applying the dual-pressure profile of E60 for tissue penetration followed by E10 for gentle cell injection. The graph displays the mean yields and viability of the cells after injection into capture fluid as indicated on the *x*-axis. The outcome of the cell counting is presented on the *y*-axis in per cent.

The in vitro injection of inert fNPs, fMPs, and isotonic liquid into pig cadaveric urethra samples indicated that pressure settings of E60 were required to position regenerative components close to or in the sphincter muscle (Figure 1, Figure 2, Figure 3, Figure 4, Figure 5, Figure 6, Figure 7 and Figure 8). At the same time, only pressure levels at or below E10 granted sufficient cell viability after waterjet applications with injection devices suitable, e.g., for applications through a cystoscope (Figure A2). Thus, a two-phase pressure profile was developed to grant a sufficient tissue penetration pressure at E60 (Figure 1, Figure 2, Figure 3 and Figure 7) followed by a gentle cell application pressure at E10 (Figure A1) [51]. With this two-phase pressure profile, porcine ADSCs and MPCs were injected into an isotonic capture fluid by a novel injection prototype (Figure 10). With ADSCs, 59 ± 12% of cells were recovered with excellent viability (98 ± 2%). With MPCs, 61 ± 20% of cells were recovered with excellent viability (97 ± 3%). These results confirmed that an excellent cell viability can be achieved by waterjet applications with the novel injection device at E10, not only with established cell lines, but also with primary cells such as pig ADSCs or MPCs (Figure 10). These results are the basis for the next level of investigations employing the novel non-invasive needle-free waterjet technology to inject bioactive components, including cells, in a large animal model of SUI therapy.

## 4. Discussion

Transurethral needle injections into the urethral sphincter complex frequently resulted in misplacement of the cells in our preclinical animal model of SUI cell therapy [39]. Based on the waterjet technology, originally developed for transepithelial injection of isotonic solutions to lift, e.g., epithelia in the gastrointestinal tract [52], we developed and refined this technology to utilise the waterjet for minimally invasive and transurethral applications. First of all, we determined the pressure levels as a function of the calibres of the devices necessary to grant sufficient throughput of active components into the tissue targeted and at the same time a high cell viability when cells were to be injected. The minimum vitality of the cells defined in the specifications was based on the information provided by the health authorities for the use of cells as advanced therapy medical products [53]. These preparatory studies suggested that wide calibres of 400 μm to 500 μm granted a sufficient transport efficacy and high cell viability at a variety of pressure levels. However, the limitations of the width of the working channels of cystoscopes used in surgical practice, for instance in urology, required a maximal outer diameter of the device of 8 Fr (= 2.67 mm) to allow transurethral surgery under visual control. Thus, injection devices with smaller diameters were needed. Consequently, waterjet prototypes with smaller calibres were developed. This required an adaptation of the protocols to pressure levels tolerable for cell injections at or below E10. These low pressure levels granted a sufficient cell viability with different cells. However, a pressure of E40 was required to transport fNPs, fMPs, and liquids across the urothelial layer. Thus, to find a practical solution to these seemingly contradictory requirements, a two-level pressure profile was developed [51,54]. This two-level pressure profile injects solvent only with a pressure of E60 for a few milliseconds to open micro pores in the tissue targeted. Within another millisecond, the pressure level is reduced to E10 to gently flush even very sensitive compounds, such as living cells, into the micropores that have just been opened [51,54].

Depending on the individual muscle composition at positions H5 and H10 of the porcine urethra, there is a fanning out of the mucosal connective tissue towards the lumen—the injection bubble—and a compression of the sphincter muscle upon waterjet injection. This phenomenon can be observed in both pressure profiles, E60 and E80, and especially upon injections at the more proximal urethral zone, H5. One cause contributing to the differences in fanning out of the tissue targeted was associated with the distinct characteristics of the sphincter muscle tissue [46,50].

The injection bubble generated in the cadaveric tissue by waterjet remained intact during the individual experiments and until the samples were frozen. This indicated that the injection hole, generated by this technology, was so narrow that the fluid applied did not leak out quickly. This is one significant advantage of this technology. Of note, bubbles were generated in the cadaveric tissue upon injections of fNPs or fMPs with needles, as used for transurethral injections, as well [55]. But these bubbles leaked out comparably fast as the injection needle punched a pinhole in the tissue.

Upon waterjet injections, the fNPs and fMPs were found in different positions relative to the urothelial tissue representing the luminal side of the urethra and the sphincter muscle, representing the outer rim of the urethra. In some cases, the fNPs and fMPs were found in the centre of the injection bubble. We consider this an artefact caused by the elastic sponge underneath the urethral tissue and the tissue elasticity itself. On the test bench, the wave of injection fluid presses the fNPs and fMPs vertically downwards through the urethral tissue. There, they oscillate in the injection bubble, and most of them form a heap in the middle of the bubble. In order to develop the regenerative potential of the cells or of other active compounds in vivo, such a condensation is not favourable.

However, upon waterjet injections of cells in the urethrae of healthy live pigs and after a follow-up of several days or weeks, this clustering of the injected compounds was not observed. By contrast, the cells were dispersed in the urethral mucosa and close to the sphincter muscle [54,56]. The improved distribution of cells through waterjet injections is another significant advantage of this new technology. It avoids repeated needle injections in the urethra, which carry a high risk of tissue injury. However, the safety and efficacy of this novel technology remain to be investigated in a SUI large animal model in future studies.

In this exploratory study we employed fresh cadaveric samples from the slaughterhouse. This inherits some advantages: The samples can be obtained at the required quantity, on a regular basis, without need of an animal study approval, and at low expenses. The disadvantages include, however, that the history of each animal remains unknown, and the time elapsed between slaughter and the experiment cannot be controlled exactly. In most cases, the samples arrived in the lab within a time span of 30 to 60 min and differences in tissue conditions were not noted. There is no indication that later injections had a noticeable impact on the outcome. In any case, the results of this study should be interpreted with care. The tissue employed in this study was prepare from young and healthy nullipara pigs. It was not altered or scarred by inflammation, mechanical stress (e.g., vaginal birth), aging or other pathologies. We, therefore, can currently not say whether this technology will prove effective in real clinical situations, or whether it will only be successful in some patients. Moreover, the cadaveric tissue used in this study no longer possesses natural tissue tension, blood flow, movement, and other parameters that are relevant in preclinical in vivo situations, and even more so in clinical situations. Furthermore, processes of inflammation or tissue healing cannot be investigated in this lab model. The study was primarily performed to examine predominantly the mechanical aspects of water jet injection of fluids and particles into the urethral tissue.

## 5. Conclusions

We conclude that quite different components can be injected by a waterjet through the urothelium using a pressure level of E40. The injected components reach the mucosal and submucosal cell layers of the urethra by limiting the waterjet injection pressure to an effect of E60. Full penetration of the urethra was avoided under these conditions. Moreover, by setting the appropriate injection pressure, the penetration depth can be varied in the tissue targeted to a certain degree. For the injection of viable cells into the urethra by waterjet, a two-phase pressure protocol facilitates targeting the tissue at levels between E40 and E60 without a high risk of full penetration. The low pressure level of E10 during the application of the bioactive compounds grants the injection of ADSCs or MPCs with excellent viability. Upon adjustment of the injection device and pressure profiles, this technology can also be adapted to other tissues, conditions, and diseases.

## Data Availability

Data and materials will be disclosed to colleagues in academia upon written, justified, and comprehendible request to the corresponding author.
